# Molecular Mechanisms and Potential Therapeutic Reversal of Pancreatic Cancer-Induced Immune Evasion

**DOI:** 10.3390/cancers12071872

**Published:** 2020-07-11

**Authors:** Li-Lian Gan, Ling-Wei Hii, Shew-Fung Wong, Chee-Onn Leong, Chun-Wai Mai

**Affiliations:** 1 School of Postgraduate Study, International Medical University, 126, Jalan Jalil Perkasa 19, Bukit Jalil, Kuala Lumpur 57000, Malaysia; lilian.gan@gmail.com (L.-L.G.); lingweihii@imu.edu.my (L.-W.H.); 2 School of Pharmacy, International Medical University, 126, Jalan Jalil Perkasa 19, Bukit Jalil, Kuala Lumpur 57000, Malaysia; cheeonn_leong@imu.edu.my; 3 School of Medicine, International Medical University, 126, Jalan Jalil Perkasa 19, Bukit Jalil, Kuala Lumpur 57000, Malaysia; shewfung_wong@imu.edu.my; 4 Centre for Environmental and Population Health, Institute for Research, Development and Innovation (IRDI), International Medical University, 126, Jalan Jalil Perkasa 19, Bukit Jalil, Kuala Lumpur 57000, Malaysia; 5 Centre for Cancer and Stem Cells Research, Institute for Research, Development and Innovation (IRDI), International Medical University, 126, Jalan Jalil Perkasa 19, Bukit Jalil, Kuala Lumpur 57000, Malaysia

**Keywords:** pancreatic cancer, immune evasion, immunoediting, immunotherapy, resistance

## Abstract

Pancreatic cancer ranks high among the causes of cancer-related mortality. The prognosis of this grim condition has not improved significantly over the past 50 years, despite advancement in imaging techniques, cancer genetics and treatment modalities. Due to the relative difficulty in the early detection of pancreatic tumors, as low as 20% of patients are eligible for potentially curative surgery; moreover, chemotherapy and radiotherapy (RT) do not confer a great benefit in the overall survival of the patients. Currently, emerging developments in immunotherapy have yet to bring a significant clinical advantage among pancreatic cancer patients. In fact, pancreatic tumor-driven immune evasion possesses one of the greatest challenges leading to immunotherapeutic resistance. Most of the immune escape pathways are innate, while poor priming of hosts’ immune response and immunoediting constitute the adaptive immunosuppressive machinery. In this review, we extensively discuss the pathway perturbations undermining the anti-tumor immunity specific to pancreatic cancer. We also explore feasible up-and-coming therapeutic strategies that may restore immunity and address therapeutic resistance, bringing hope to eliminate the status quo in pancreatic cancer prognosis.

## 1. Introduction

Pancreatic cancer is one of the deadliest malignancies known to mankind. The Global Cancer Statistics 2018 (GLOBOCAN) ranks pancreatic cancer as the seventh leading cause of cancer death in both males and females, with an estimated 459,000 cases and 430,000 deaths in 2018 globally [1]. With an estimated five-year survival rate of 10% or less [2], pancreatic cancer is the cancer with the highest incidence-to-mortality ratio among all solid tumors [3]. Pancreatic cancer has also surpassed breast cancer and will soon take over colorectal cancer as the second leading cause of cancer mortality before the end of 2030 [4]. Pancreatic ductal adenocarcinoma (PDAC) constitutes up to 90% of all pancreatic cancers [5]. Despite advancement in surgical techniques and treatment strategies, there has not been much improvement in the survival outcomes of the patients. Delayed diagnosis is the major problem, contributed to by the absence of effective screening methods and insidious clinical presentation of PDAC. Patients diagnosed with PDAC have grim prognosis. Only a handful of patients with pancreatic cancer are able to undergo curative surgical resection, coupled with adjuvant chemotherapy, albeit the majority of those who underwent surgery eventually develop recurrence and succumb to the disease [6]. Systemic chemotherapies are the current mainstay but most of these treatments have yet to achieve the desirable clinical outcomes [7]. Hence, there is a desperate need to uncover the in-depth pathophysiology behind pancreatic cancer, and thus to design an effective diagnosis and treatment.

Recently, the discovery of cancer and immune cell interaction in the tumor microenvironment (TME) has generated much excitement. Immune cells of both innate and adaptive immune systems play important roles in the initiation and progression of cancer [8,9]. Suppression of immune rejection leads to enhanced tumorigenesis and metastasis [10,11]. In many cancer types, clinical decisions are made based on robust histopathological criteria; however, this is not applicable to pancreatic cancer subtypes [12]. Pancreatic cancer that appears morphologically identical does not always share similar clinical features and therefore responds variably to therapy. Cancer classification, including PDAC, is only beneficial when the specified subtype reflects clinical relevance to guide therapeutic management and predict disease outcomes. 

To effectively improve the prognostic outcomes of PDAC patients, these newly found cancer–immune interaction must be translated to clinical practice. We believe that only when the identified disease subtypes are well-established with a clear predictive model in place, coupled with the comprehensive molecular and immune characterization of each patient, precision medicine can be optimized to reach its fullest potential in treating PDAC. In the present paper, we discuss the current literature supporting PDAC-induced immune suppression via innate and adaptive evasion, and comprehensively reviewing potential therapeutic strategies that may overcome these intrinsic immunosuppressive mechanisms in PDAC.

## 2. The PDAC Intrinsic Mechanisms of Immune Evasion

**Figure 1:**
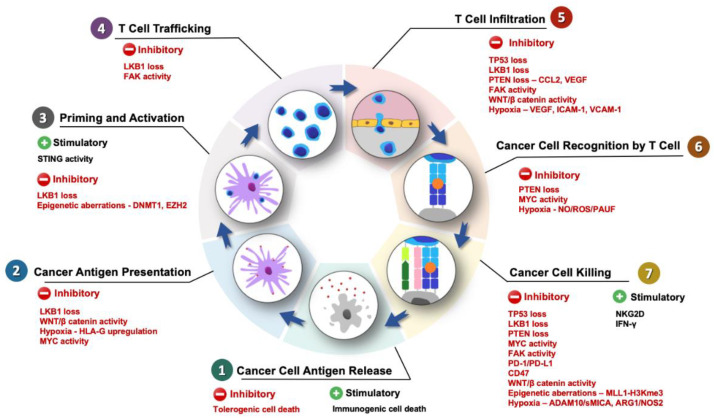
Stimulatory and inhibitory factors that may affect the cancer immunity cycle in pancreatic ductal adenocarcinoma (PDAC). The anti-tumor immune response cycle starts with cancer cell antigen release (Step 1), whereupon the apoptosis of cancer cells (gray cells) takes place and cancer cell antigens (red small dots) are released and subsequently picked up by dendritic cells (purple cells). Antigen-presenting cells, mainly dendritic cells, can then process the cancer-specific antigens and present them to T helper cells (Step 2), allowing for the priming and activation (Step 3) of the immune cells (blue circular cells). Once activated, the immune cells traffic to the tumor site (Step 4) and infiltrate the tumor (Step 5). Within the tumor microenvironment (TME), cancer cell recognition (Step 6) by the T cell eventually leads to cancer cell killing (Step 7). LKB1, liver kinase B1; MYC, oncogenic myelocytoma; HLA, human leukocyte antigen; STING, stimulator of interferon genes; DNMT, DNA methyltransferase 1, EZH2, enhancer of zeste homolog 2; FAK, focal adhesion kinase; PTEN, phosphatase and tensin homolog; NO, nitric oxide; ROS, reactive oxygen species; PAUF, pancreatic adenocarcinoma upregulated factor; NK2D, natural killer group 2D receptor; IFN, interferon; PD-1, programmed cell death protein 1; PD-L1, programmed death-ligand 1; CD47, cluster of differentiation 47; H3K4me, H3K4 trimethylation; ADAM10, A Disintegrin and Metalloproteinase Domain 10; sMICA, soluble MHC class I-related molecule A; HIF1A, hypoxia-inducible factor 1-alpha; CCL2, Chemokine ligand 2; COX2, cyclooxygenase 2; PGE2, prostaglandin E2; VEGF, vascular endothelial growth-factor; ARG1, arginase 1; NOS2, nitric oxide synthase 2.

Tumors with a high degree of T-cell infiltration are regarded as T-cell-inflamed or “hot” tumors, whereas tumors with low levels of T-cell infiltration are termed as non-T-cell-inflamed tumors or “cold” tumors [13]. PDAC falls in the latter category, hence explaining its lack of success in immunotherapy [10,11]. Tumor cell-intrinsic aberrations can lead to a “cold” tumor by excluding cells that can mount an effective anti-tumor response or by attracting immunosuppressive population to the TME [14]. The reported immune cell types of adaptive immune system comprise of B cells, cytotoxic T lymphocytes (CTLs), memory T cells, helper T cells and regulatory T (Treg) cells, whereas those from innate immune system consist of macrophages, dendritic cells, mast cells, natural killer (NK) cells and myeloid-derived suppressor cells (MDSCs) [11,15]. Cells from both innate and adaptive immune systems have been demonstrated to play important roles in the initiation and progression of cancer. This can happen via the suppression of immune rejection, leading to enhanced tumorigenesis and metastasis [10,16]. Chen and Mellman postulated that the process of immunity generation in cancer as an ongoing cyclical process (Figure 1) [17]. The machinery of this process frequently leads to the accumulation of immune-stimulatory factors that act to amplify and broaden the T-cell responses. This is counterbalanced by inhibitory factors, acting as an immune regulatory feedback mechanism, either halting the development or limiting the immunity. The cancer immunity cycle consists of seven consecutive steps: dead cancer cells release neoantigens, which are then captured by antigen presenting cells (APCs); APCs such as dendritic cells then present the captured antigens on the major histocompatibility complex (MHC) molecules to T cells, leading to the priming and activation of effector T-cell responses. In parallel, a chemokine gradient exists to guide the activated T-cell infiltration to the tumor site. As a result of T-cell receptor (TCR) and neoantigen–MHC complex interaction, the killing of cancer cells occurs [17].

Recently, Spranger et al. introduced the concept of innate versus adaptive immune evasion [18]. Innate immune evasion is regarded as the major mechanism behind most cases of primary resistance against checkpoint blockade therapy. Essentially, T cells and other anti-tumor immune cells are excluded from the TME in the absence of immunoediting. On the other hand, adaptive immune evasion involves immunoediting, allowing the tumor to establish equilibrium with existing T-cell infiltrates. It encompasses patients who initially experience clinical response and then develop secondary resistance under immune selective pressure [18]. In principle, relatively “cold” tumors such as pancreatic cancer could arise due to disruption at any major step within the framework of the cancer immunity cycle.

## 3. Molecular Mechanisms Involved in Innate Immune Evasion of PDAC

### 3.1. Tumor Protein p53 (TP53)

The tumor suppressor TP53 is a key regulator of cell cycle progression. The mutation of TP53 is found in approximately up to 70% of PDACs [19]. In the presence of multiple stressors that will trigger uncontrolled cell proliferation, it safeguards against cell division by inducing cell death or senescence [20]. It is also evident that immune evasion happens early during tumorigenesis as TP53 is often lost at an early stage in the process of cancer development. Consistent with this finding, several studies also showed cooperation between Kirsten rat sarcoma viral oncogene homolog (KRAS) and TP53 mutation in immune evasion, where increased expression of CXCR3/CCR2-associated chemokines and granulocyte-macrophage colony-stimulating factor (GM-CSF) occurs in various PDAC models [21,22,23]. This leads to the accumulation of immunosuppressive Gr1^+^CD11b^+^ MDSCs and attenuation of CD4^+^ T helper cells, besides the prevention of CD8^+^ T-cell infiltration and cytolytic activities [21,22]. A recent study also showed that the concurrent activation of KRAS and loss of TP53 can promote immune tolerance not only through myeloid cell recruitment, but also through Treg cell selection [23]. Moreover, Treg cells of TP53-harboring PDAC display heightened suppressive capability compared to their wild type counterparts [23].

Cellular senescence is triggered by TP53, followed by the rapid elimination of the senescent tumor cells by NK cells [24]. For example, TP53 restoration in a murine liver cancer model showed an increase in CCL2 that can upregulate NKG2D, thus promoting NK cell activation and recruitment [24]. Pharmacological TP53 reactivation led to improved NK-mediated breast cancer cell lysis. Reactivation of TP53 via pharmacological treatment in patients with lymphoma and melanoma could induce systemic anti-tumor immunity and immunogenic cell death [25]. Indeed, many solid tumors have also shown reinvigoration of anti-tumor response secondary to TP53 restoration. Taken all these together, it is possible that TP53 reactivation strategies may be of useful to eradicate PDAC, which warrants further investigations.

### 3.2. Liver Kinase B1 (LKB1)

The tumor suppressor liver kinase B1 (*LKB1*) encodes a serine/threonine kinase that phosphorylates AMP-activated protein kinase (AMPK), a critical modulator of cell proliferation and polarity [26]. The reduced LKB1 expression is associated with poor survival in PDAC patients [27]. In a general immunological context, LKB1 plays a multi-faceted role in regulating the development, proliferation and activation of T effector and Treg cells. Other observations related to LKB1 loss in the TME are: (1) increased numbers of Tregs; (2) the overexpression of T-cell inhibitory markers, such as PD-1, T-cell immunoglobulin mucin-3 (TIM-3), lymphocyte-activation gene 3 (LAG-3) and cytotoxic T-lymphocyte-associated antigen 4 (CTLA-4); and (3) a reduction in tumor-infiltrating lymphocytes (TILs), which also exhibit elevated markers of T-cell exhaustion [28]. 

Stimulator of interferon gene (*STING*) has recently emerged as an important immunomodulator linked to LKB1 expression in *KRAS*-mutant cancer [29]. Specifically, LKB1 inactivation is associated with STING suppression via the hyperactivation of epigenetic silencing enzymes DNA methyltransferase 1 (DNMT1) and enhancer of zeste homolog 2 (EZH2) activity due to elevated levels of S-adenylmethionine (SAM) substrate, and partly by DNMT1 upregulation [29]. Yes-associated protein (YAP) is one of the key downstream regulators in the Hippo pathway, which is upregulated in PDAC. Recently, the YAP transcriptional co-activator has been identified as a downstream target of the *LKB1* tumor suppressor [30]. LKB1 phosphorylates YAP, leading to nuclear exclusion and degradation of YAP. Intriguingly, this process is independent of canonical YAP kinases (large tumor suppressor kinase 1/2, LATS1/2) and metabolic downstream targets of LKB1 (AMPK and mTORC1), and is directly reflective of LKB1-induced morphological transformation [30]. YAP abrogation was shown to deplete MDSCs, increase antigen-presenting macrophage infiltration, and cause T-cell reactivation [31].

### 3.3. Epigenetic Aberrations

Epigenetic aberrations can occur as a result of genetic, environmental and metabolic influences [32]. In a pan-cancer involving The Cancer Genome Atlas (TCGA) data analytical study, it was reported that global methylation loss can promote the immune evasion of tumors with high mutation and copy number load, hence genomic demethylation implicates epigenetic modulation as a part of regimen for precision immunotherapy [33]. PDAC is associated with immune tolerance, a state that is mediated by complex shifts in the number, phenotype and function of multiple immune cells [34]. Immunogenic cell death (ICD) is a critical pathway to overcome the immune tolerance in PDAC, as it can induce the emission of damage-associated molecular patterns (DAMPs) and restore the three main signals that activate anti-tumor T cells, including increased antigen presentation following cell death; co-stimulation from matured and recruited APCs; and cytokine production from tumor cells and APCs [35]. Besides, there is also evidence linking the epigenetic aberrations with the expression of PD-L1. Specifically, H3K4 trimethylation (H3K4me3) is enriched in the CD274 (PD-L1) promoter of pancreatic tumor cells. Mixed lineage leukemia protein-1 (MLL1), a histone methyl transferase can bind directly to the CD274 promoter to catalyze H3K4me3, and upregulate the transcription of PD-L1 [36]. Hence, targeting epigenetic aberrations in PDAC may potentially improve the sensitization and priming of the host immune responses, thus improving the efficacy of immunotherapeutic agents. 

### 3.4. Phosphatase and Tensin Homolog (PTEN)

PTEN is a potent tumor suppressor that antagonizes oncogenic signaling and maintains genomic stability [37]. It functions to antagonize the catalytic activity of phosphoinositide 3-kinase (PI3K), thus contributing substantially to the downstream effects of the PI3K/AKT/mTOR signaling pathway, including tumorigenesis, metabolism and immunity [38]. Transcriptomic analyses of murine PDAC models combining *KRAS* and *PTEN*-loss showed marked NF-κB activation and its cytokine network, accompanied by strong stromal activation and immunosuppressive cell infiltration [39]. 

Considering that PTEN loss affects the immunophenotype of various other cancers, the elucidated immunomodulatory mechanisms may be applicable in providing important insights relevant to PDAC. Similar to PDAC, it appears that several melanoma, glioblastoma and gastric cancer studies have reported the link between PTEN loss and PI3K-pathway-driven immunosuppression [40,41,42]. Studies have shown that the murine model bearing *PTEN*-deleted melanoma tumors and PTEN loss leads to T-cell exclusion via two mechanisms: firstly, the inhibition of CD8^+^ T-cell killing and decreased effector T-cell infiltration through the expression of immunomodulatory cytokines, including CCL2 or vascular endothelial growth-factor (VEGF); secondly, reduced tumor immunogenicity by the inhibition of the autophagy pathway responsible for immunogenic cell death [43]. Although the majority of immune-related functions of PTEN can be linked to its canonical activity that opposes PI3K signaling, further exploration of the PTEN activity in T cells and pancreatic tumor cells are warranted to allow for the identification of novel immunotherapeutic targets. 

### 3.5. WNT/β-Catenin

The canonical Wingless related integrated (WNT)/β-catenin signaling cascade is well known for its significance in stem cell renewal. Its role as a key driver of oncogenesis and cancer progression in many different malignancies is also well characterized [44]. WNT/β-catenin overactivity in PDAC can result from genetic perturbations of several WNT-regulating genes, such as adenomatous polyposis coli (APC) hypermethylation [45], multiple endocrine neoplasia (MEN1) inactivation [46], ring finger protein 43 (RNF43) inactivation [47], WNT-inhibitory factor 1 (WIF1) hypermethylation [48], and Dickkopf-1 (DKK1) dysregulation [49]. A recent pan-cancer in silico integrative analysis of the TCGA data concluded that WNT/β-catenin pathway activation is frequently associated with poor spontaneous T-cell infiltration across most human cancers [50]. Specifically, a significant inverse correlation (*p* < 0.001) between β-catenin levels in PDAC cells and T-cell-inflamed gene expression was noted [50], signifying that the impaired T-cell-mediated immunity in PDAC is partly attributed to WNT-signaling activity. Gene-expression analysis of the RNA-seq dataset of 143 PDAC patients from the PACA-CA cohort of International Cancer Genome Consortium (ICGC) revealed the presence of increased WNT activation with a peculiar, tumor tolerogenic immune microenvironment among subjects with nodal involvement [51]. 

A separate study demonstrated a significant negative correlation between CD103^+^ DC infiltration and nuclear β-catenin (*p* < 0.05) was observed. The Batf3-dependent CD103^+^ DC is a specific dendritic cell subset, which plays a crucial role in mounting an effective T-cell response via cross-presentation. Cross-presentation is a critical step in priming the anti-tumor T-cell response via the presentation of exogenous antigens on MHC class I molecules to naive CD8^+^ T cells [52]. Together, these data suggest that targeting the WNT/β-catenin pathway is a promising therapeutic approach in helping to induce a T-cell-inflamed TME and augment effectiveness of checkpoint blockade therapies.

### 3.6. Hypoxia

Hypoxia is a common metabolic aberration occurring as a result of rapid tumor cell proliferation and inadequate angiogenesis in various cancers [53]. VEGF overexpression is a frequent finding in human pancreatic tumor biopsies, reflecting the relevance of hypoxia in the PDAC microenvironment, impacting tumor growth dynamics and confer immunotherapeutic resistance [54,55]. Hypoxia-induced production of VEGF, together with other cytokines, including IL-10, IL-6 and G-CSF can hinder the maturation of DCs, reducing the fully-fledged DC population [56]. Moreover, matrix metalloprotease type-9 (MMP-9) produced by tumor-associated neutrophils (TANs) and macrophages in the pancreatic TME can further exacerbate VEGF-mediated immunosuppressive effects [57]. 

In addition to this, HIF1A together with A Disintegrin and Metalloproteinase Domain 10 (ADAM10) and soluble MHC class I-related molecule A (sMICA) are upregulated during hypoxia, and all of which can reduce the expression of natural killer group 2D (NKG2D) receptor on NK cells, allowing tumor cells to escape from immune surveillance and NK cell-mediated lysis [58,59]. The nitric oxide-cyclic guanosine monophosphate-protein kinase G axis is another pathway that is markedly inhibited by hypoxia, and its reactivation was shown to promote sMICA and NKG2D expression and NK cell cytotoxicity, diminishing hypoxia-mediated immunosuppression of PDAC cells [58]. In a hypoxic TME, tumor-induced macrophage polarization (from M1 to M2) occurs via increased nitric oxide (NO) and decreased arginine in hypoxia, as reflected by transcript levels of arginase 1 (ARG1) and nitric oxide synthase 2 (NOS2) in PDAC models [55,60]. Murine PDAC models showed that the increase in nitric oxide (NO), arginase and reactive oxygen species (ROS) can be induced by PDAC upregulated factor (PAUF) from tumor cells to prevent T-cell receptors from accurately recognizing antigens, thus impairing their function [55,61]. 

### 3.7. Focal Adhesion Kinase (FAK)

The presence of numerous immunosuppressive immune cells in a densely fibrotic stroma in PDAC microenvironment has been well-characterized by various studies [62,63]. Analyses of human pancreatic tumor tissues have revealed that T cells can infiltrate pancreatic lesions, and similar to other cancer types, T-cell infiltration in PDAC correlates with a favorable response to immune checkpoint blockade therapy [64]. Focal adhesion kinases (FAK) are non-receptor tyrosine kinases that regulate various cell processes, such as adhesion, migration and proliferation. FAK have been implicated in diseases where fibrosis predominates, and their upregulation has also been observed in a number of cancers, including PDAC [65]. Up to 80% of PDAC samples overexpressed FAK1 and exhibited higher levels of activated phosphorylated FAK1 (p-FAK1) than normal pancreatic tissues, together with lower levels of CD8^+^ T-cell infiltration. The assessment of overall survival based on these two markers revealed that high p-FAK1 and CD8^+^ T-cell infiltration correlated with poorer outcomes. Noticeably, FAK inhibitor treatment led to a lower level of phosphorylated-STAT3 in murine PDAC tumor cells [63]. This is relevant, considering that activated STAT3 signaling in PDAC epithelium has been demonstrated to mediate tumor progression by promoting fibrosis and stromal stiffening [66]. Altogether, these findings provide a solid basis to implicate fibrotic stroma as a key player in promoting an immunosuppressive TME.

### 3.8. MYC Oncogene

The *MYC* oncogene regulates a wide array of gene products in cell proliferation, apoptosis, growth and differentiation; its role in pancreatic cancer tumorigenesis and invasiveness is well established [66,67,68]. A recent study demonstrated that in vivo acute activation of MYC in *KRAS^G12D^*-driven pancreatic intraepithelial neoplasm (PanIN) alone can trigger the immediate release of instructive signals capable of driving transition to PDAC alongside their characteristic stromal features [68]. Through a direct *MYC*-dependent transcription, PD-L1 is expressed autonomously by PanIN epithelial cells to exclude CD3^+^ T cells [68]. 

MYC is reported to promote tumor cell-intrinsic immune evasion by mediating the overexpression of PD-L1 and CD47 on cancer cells [69]. Both PD-L1 and CD47 are important “don’t find me” and “don’t eat me” signaling molecules, respectively, which work synergistically in a coordinated fashion to evade the innate and adaptive immunity [69]. The CD47 anti-phagocytic ligand is highly expressed in a majority of primary pancreatic cancer stem cells (CSCs) and targeting CD47 on PDAC cells demonstrated changes in the behavior of immunosuppressive resident tumor-associated macrophages (TAMs) to inhibit tumor growth instead [70]. 

Overall, the immunomodulatory effects of MYC oncogene in PDAC mainly happen via increased expression of PD-L1 and CD47 to evade both innate and adaptive immune responses, thus favoring tumor growth. The in vitro and in vivo studies on anti-MYC or anti-CD47 treatment have proven its effectiveness in treating PDAC, albeit not as potent as a monotherapy modality [70], yet warrants the need for further pre-clinical studies to determine the ideal combination and optimum dosage for maximal therapeutic efficacy and safety for patients.

## 4. Immunosuppression via Adaptive Immune Evasion in PDAC

As opposed to the innate nature of the earlier described pathways, the adaptive mechanisms are generally acquired as responses to selective pressure exerted by the host’s immune response or from treatment. Cancer immunoediting is a tumor sculpting action of the immune system intended to specifically target and eliminate highly immunogenic cancer cells; nonetheless, the trade-off in this process is the exertion of “Darwinian-like” selective pressure on developing cancer, ultimately favoring the outgrowth of cancer cells with poor antigenicity [71,72]. In fact, the three Es of cancer immunoediting, namely elimination, equilibrium and escape, aptly reflects the development of pancreatic cancer and immune landscape of its TME [73]. Likewise, it is plausible that a similar immunoediting process could lead to secondary resistance among patients treated with immunotherapies in the clinical setting [74,75]. 

Conversely, numerous studies on PDAC have proposed the concept of immunological ignorance or immune-privileged status, where T-cell exclusion manifests in the absence of immunoediting, suggesting that immunoediting may result from the poor antigenicity of PDAC cells, not otherwise [76,77]. Furthermore, “cold” tumors such as PDAC were observed to undergo immunosuppression as an early event, where T-cell reactivity fails to fully unfold, likely driven by poor T-cell priming [77]. Another intrinsic determinant of immunogenicity is the role of Janus kinases (JAKs) in the downstream signal transduction of interferon (IFN) receptors, whereby the loss of JAK function can disrupt IFN signaling [78,79]. In cell lines bearing *JAK1* and *JAK2* mutations, diminished IFN-γ signaling leads to the reduced expression of MHC-I molecules and a lack of recognition by CD8^+^ T cells. Another intrinsic ability of the mutant tumor cells that allowed their accelerated growth is the development of resistance towards IFN-γ-mediated cell growth arrest. Altogether, these data reflect the presence of multiple inhibitory pathways that cripple T-cell immunogenicity and the mechanistic basis underlying the failure of immune checkpoint blockades in treating PDAC, justifying the needs to develop sound therapeutic strategies to enhance the antigenicity and immunogenicity of PDAC. 

## 5. Therapeutic Targets to Overcome Immune Evasion in PDAC

**Figure 2:**
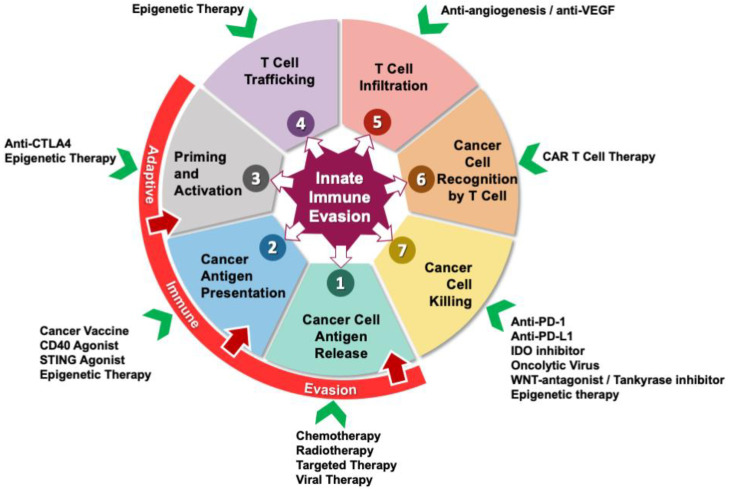
Cancer-immunity cycle can be affected by both innate and adaptive immune evasion mechanisms. Innate immune evasion (dark purple star in the center) is intrinsically driven by pancreatic tumor cells, it can disrupt any steps in the cancer-immunity cycle through various pathways described earlier in Figure 1, whereas the adaptive counterpart (red arc over steps 1 to 3) primarily evades the immunosurveillance of the host via immunoediting and impaired priming of T cells. Conventional therapies such as chemotherapy and radiotherapy (RT), along with newer strategies such as targeted therapy and viral therapy can help in causing cancer cell antigen release (step 1). Cancer antigen presentation (step 2) can be augmented by the use of cancer vaccines, CD40 agonist, stimulator of interferon genes (STING) agonist and epigenetic therapy. Consistent with the effect of epigenetic aberrations on many steps in the cycle, epigenetic treatment can also be employed to target the priming and activation of the T cells (step 3), T-cell trafficking (step 4), and cancer cell killing (step 7). Checkpoint blockade agents including anti-CTLA1, anti-PD-1, and anti-PD-L1 inhibitors can enhance priming/activation and initiate cancer cytolysis, respectively. Targeting anti-angiogenesis via anti-VEGF treatment can help improve T-cell infiltration (step 5) into the tumor microenvironment (TME). CAR T-cell administration is a promising strategy to improve cancer cell recognition by T cells (step 6) in the TME. Numerous strategies besides immune checkpoint blockade therapy can augment cancer cell killing (step 7) by effectors cells within the TME, such as indoleamine 2,3-dioxygenase (IDO) inhibition, oncolytic virus, tankyrase inhibition to antagonize WNT signaling, and epigenetic modulating approaches.

The immune system is capable of recognizing and eliminating tumor cells; cancer usually develops as a result of failed immune surveillance. Immunotherapy has been a game-changer in the management of various solid cancers [80,81,82,83]. Immunotherapeutic approaches include immune checkpoint inhibitors, cancer vaccines, oncolytic viruses, and adoptive cell transfers have been attempted in PDAC. Pancreatic cancer cell-intrinsic immune evasion machinery can affect the cancer-immunity cycle differently: innate immune evasion that disrupts any steps in the anti-tumor immunity cycle, whereas adaptive immune evasion involves immunoediting or defective priming of anti-tumor immune response (Figure 2). Although pancreatic cancer cells possess the intrinsic ability to weaken anti-tumor immune responses, a plethora of promising therapeutic strategies have been discovered and developed to counterattack these mechanisms. 

### 5.1. Immune Checkpoint Inhibitors

PD-1 is expressed by effector T cells, Treg cells, B cells and NK cells and binds to the PD-L1 ligand [84]. PD-1 monoclonal antibodies (mAb) have achieved great clinical success for a variety of solid tumors, including melanoma, NSCLC, urothelial carcinoma and renal cell cancer [85,86]. Numerous studies have reported that high PD-L1 expression in PDAC is associated with poor prognosis [87,88]. However, results from a phase I trial of anti-PD-L1 therapy used in pancreatic cancer showed no evidence of improved clinical response [82].

Moreover, a blockade of CTLA-4, a co-inhibitory receptor expressed on CD4^+^ and CD8^+^ T cells, can enhance anti-tumor immunity [89]. Ipilimumab (anti-CTLA-4) was shown to improve overall survival in melanoma patients [83]. Unfortunately, phase II studies for anti-CTLA-4 agents such as ipilimumab [90] and tremelimumab [91] showed no clinical advantage in PDAC patients. Otherwise, combinatory approaches incorporating immune checkpoint blockade, seem promising and warrant more investigations. A phase II, randomized trial evaluating durvalumab monotherapy versus durvalumab plus tremelimumab as a second-line treatment for PDAC showed a disease control rate of less than 10% in both arms [90]. 

Evidently, cancers with high mutational load such as melanoma, lung cancer and bladder cancer often respond better to checkpoint inhibition than those with lower mutational burden, such as pancreatic carcinoma [92]. Approximately only 1% of PDAC harbors microsatellite inactivation, providing a plausible explanation for the low response rate to immune checkpoint inhibitor therapy [93]. Before considering tumor mutational burden rate as a predictor of immunotherapeutic response in PDAC, a well-planned characterization assay to determine mutation in cancers is needed to establish the clinical applications of this potential biomarker.

Accumulating evidence suggests that immune checkpoint inhibitors have limited potential to produce considerable clinical advantages in treating PDAC as single agents. Thus, numerous efforts have been made to develop combinatory strategies [94,95,96] with several other modalities to regulate the anti-tumor immune response in PDAC, either by: (1) targeting the immunosuppressive TME, or (2) improving T-cell priming—where “cold” tumors are turned into “hot” ones [11].

Preclinical studies have elucidated the immunosuppressive roles of TAMs and MDSCs in reducing the survival of patients with PDAC by mediating tumor progression, metastasis and conferring resistance towards chemoradiotherapy [64,94]. The inhibition of colony-stimulating factor 1 receptor (CSF1R), which is ubiquitously expressed by TAMs and MDSCs, have been shown to improve the efficacy of anti-PD1 or anti-CTLA4-based immunotherapy in pancreatic cancers by depleting TAMs and reprogramming the activity of the remaining macrophages [94]. A two-part Phase I trial (NCT02777710) involving the combination of PLX-3397 (anti-CSF1R) with durvalumab (anti-PD-L1 Ab) has shown promising clinical benefits during its dose-finding escalation part in patients with advanced pancreatic and colorectal cancers, and more results are yet to be released from its second enrolment in the expansion cohorts, which was just completed in January 2019 [97]. Meanwhile, another phase I clinical trial is currently evaluating the combination of IMC-CS4 (CSF1R mAb) with pancreatic cancer vaccine (GVAX) and pembrolizumab (anti-PD-1 mAb) in patients with borderline resectable pancreatic cancer (NCT03153410).

Mitogen-activated protein kinase (MAPK) kinase (MEK) inhibition in combination with PD-L1 blockade is a promising strategy to enhance anti-tumor immune responses [98,99,100]. Although MEK inhibition was shown to block naïve CD8^+^ T-cell priming, it protected the tumor-infiltrating CD8^+^ T cell from cell death driven by chronic TCR stimulation with sparing of cytotoxic activity, thus resulting in a synergistic and durable tumor regression, given that either agent alone was only modestly effective [98]. Let us not forget that MEK inhibition has promising anticancer effects [101,102]. NCT03193190 is an ongoing phase Ib/II clinical trial evaluating the combination of cobimetinib (MEK inhibitor) with atezolizumab (anti-PD-L1) for patients with metastatic PDAC. Another clinical trial (NCT03637491) investigating avelumab (anti-PD-L1) and binimetinib (MEK inhibitor) in combination for patients with PDAC is also underway.

Although not yet fully understood, autophagy has been recognized as a contributing factor of pancreatic tumorigenesis and autophagy inhibition was shown to cause tumor regression through various tumor-intrinsic and -extrinsic mechanisms [103,104]. More recently, autophagy inhibition with chloroquine has been shown to synergize with dual immune checkpoint blockade therapy (anti-PD1 and anti-CTLA4) in PDAC, through the restoration of surface MHC-I levels, improved antigen presentation and enhanced CD8^+^ T-cell responses [105]. In melanoma and colorectal carcinoma, the inhibition of autophagy-related protein and vacuolar protein sorting 34 (Vps34) kinase activity have been shown to turn “cold” tumors into “hot” tumors, rendering the tumor cells more susceptible to immune checkpoint inhibition [106]. Thus far, clinical trials involving the use of autophagy and immune checkpoint inhibitors are pending to facilitate the development of this combination in treating PDAC.

Studies investigating the combination of radiotherapy and immunotherapy have demonstrated a synergistic effect, favoring the anti-tumor immune response including PDAC [98,107]. Specifically, Azad et al. [107] have reported that RT and chemotherapy upregulate PD-L1 in a JAK/STAT-dependent manner, plus the addition of anti-PD-L1 to high RT dose with chemoradiotherapy improved tumor response and decreased the formation of liver metastases in PDAC. Moreover, the intratumoral milieu is shifted away from the infiltration of immunosuppressive MDSCs and Tregs towards the infiltration of activated CD8^+^ T cells. Building on the same concept, a phase I/II study is currently investigating the efficacy of immune checkpoint inhibitors (tremelimumab and/or MEDI4736) with RT for patients with metastatic pancreatic cancer who previously received systemic chemotherapy (NCT02311361). Another randomized phase II study (NCT02866383) is currently ongoing to determine the efficacy and safety profile of nivolumab or nivolumab plus ipilimumab administered to high dose RT for patients with metastatic PDAC.

### 5.2. Cancer Vaccines

Cancer vaccines are designed to stimulate the host immune system to recognize and destroy tumor cells using specific effector and memory T cells. GVAX is a whole-cell vaccine developed from genetically engineered pancreatic tumor cells that secrete GM-CSF. GVAX has been tested in combination with different substances, including with a Listeria vaccine expressing mesothelin but results were disappointing [108]. Another whole cell vaccine, algenpantucel-L, which consists of irradiated cancer cells expressing alpha-1,3-galactosyltransferase, was combined with radiochemotherapy and administered as adjuvant therapy in a phase II study. The study reported promising results, with a median disease-free survival of 17.3 months in 60 PDAC patients [109]. However, the development of this compound was halted when it failed to meet its primary endpoint in a confirmatory phase III trial (IMPRESS) [110]. On a side note, non-whole cell vaccines such as GV1001 [111] or PANVAC-V [112] also do not provide a statistically significant clinical advantage over standard therapies in pancreatic cancer. Following the failure of cancer vaccines, current ongoing trials are exploring the strategy of combining vaccines with immune checkpoint inhibitors [5].

### 5.3. Adoptive Cell Therapy

Adoptive cell therapy manipulates the anti-tumor properties of lymphocytes to eradicate primary and metastatic tumor cells, making it a potentially powerful immunotherapy modality [113]. The chimeric antigen receptor (CAR) T cells are produced from autologous T cells isolated from patients’ peripheral blood; these cells then undergo ex-vivo transduction to express a CAR specific for a tumor antigen of choice, followed by expansion, before being re-infused to the patient as a therapy [114,115]. A great number of clinical trials utilizing CAR-T cells on pancreatic cancer patients has been undertaken to evaluate its safety and efficacy. The majority of these trials are in early stages of recruitment, albeit some have been completed with initial reports available [115]. Preliminary results from a phase I study evaluating the use of anti-mesothelin-specific CAR-T cells show promising findings [116]. A different modality, Claudin 18.2 (CLDN18.2), is a gastric-specific membrane protein targeted to treat gastric cancer and other cancer types. Anti-CLDN18.2 CAR investigation on gastric and pancreatic cancers under the trial reported some objective responses with no overt toxicities [117,118]. Nevertheless, the unique challenges presented by the TME and immune landscape of pancreatic cancer may be the reason behind the lack of significant responses in recent trials, which warrant further optimization of treatment strategies involving adoptive cell therapy.

### 5.4. Oncolytic Virus

As the name suggests, oncolytic virus therapy involves the use of native or engineered viruses that selectively replicate in cancer cells that ultimately destroy them [119]. Theoretically, viral-inoculation-induced immunomodulation can transform an immunologically “cold” tumor into a “hot” tumor, as in the case of pancreatic cancer [120]. Moreover, oncolytic virus infection can increase the expression of PD-L1 receptors on tumor cells [121]. In a recently published phase Ib trial incorporating intravenous reolysin and pembrolizumab for patients with metastatic pancreatic adenocarcinoma, treatment was well-tolerated with durable efficacies [122]. A follow-up phase II study using pelareorep and pembrolizumab on patients with advanced pancreatic cancer is underway (NCT03723915). Similarly, preliminary results from an ongoing trial combining oncolytic virus, canerpaturev (C-REV) with standard chemotherapy, and gemcitabine with nab-paclitaxel demonstrated a favorable safety profile and encouraging anti-tumor activity in Japanese patients with unresectable pancreatic cancer [123].

Ideally, an oncolytic virus can lead to optimal tumor eradication if there is widespread and diffuse viral distribution after inoculation [124]. Intra-tumoral administration of oncolytic virus in pancreatic cancer is challenging. The extracellular matrix of pancreatic cancer, with its dense fibrotic stroma and high interstitial fluid pressure, acts as an effective barrier to viral dissemination, limiting viral spread at the vicinity of the injection site [125]. The intravenous administration of oncolytic virus has its fair share of difficulties, amongst them are neutralizing antibodies, complement cascade, liver/spleen sequestration, and sub-optimal extravasation from tumor-associated blood vessels [126]. Strategies employed to solve these problems include serotype switching, host immunosuppression, PEGylation or viral shielding with polymer coating, and the use of carrier molecules that deliver virus directly to the tumor [127,128,129].

### 5.5. CD40

CD40 is a cell surface molecule that belongs to the tumor necrosis factor (TNF) receptor family. It is diversely expressed by immune cells, epithelial cells, and a wide range of tumor cells [130]. CD40 is well-known for its remarkable role in immune regulation and homeostasis [131]. Early experimental studies using CD40 agonist (CP-870,893) plus gemcitabine demonstrated encouraging results in treating advanced PDAC [132]. This combination was shown to cause pancreatic tumor regression by enhancing the accumulation of tumor-suppressive macrophages. Currently, there are six ongoing clinical testing on different CD40 agonist monoclonal antibodies. Evidence suggests that it is unlikely for CD40 monoclonal antibodies to exhibit substantial single-agent anti-tumor activity in PDAC patients; further studies are thus required to develop effective combinations of CD40 agonist therapy with chemotherapy, RT or other forms of immunotherapy [133]. 

### 5.6. Indoximod

Indoleamine-2,3-dioxygenase (IDO) is an intracellular enzyme that leads to the immune escape of tumor cells through the depletion of tryptophan [134]. Indoximod, an IDO pathway inhibitor, has been demonstrated to interfere with multiple targets within the IDO pathway [135]. Evidence of synergy between IDO pathway inhibition with indoximod and chemotherapy has been shown in preclinical models [136]. A phase 1 trial combining docetaxel and indoximod has also reported evidence of clinical activity against metastatic solid tumors with a good safety profile [137]. Other trials evaluating the combination of indoximod with standard of care chemotherapy regimens, including a trial evaluating gemcitabine and nab-paclitaxel chemotherapy for patients with metastatic pancreatic cancer, consistently proved to be safe and efficacious [135,138].

### 5.7. Epigenetic Therapy

Histone deacetylases (HDAC) are enzymes that act in tandem with their counterpart histone acetyltransferases (HAT) to regulate the acetylation status of histones and other intracellular substrates [139]. Histone acetylation represents the open chromatin configuration and active state; deacetylation by HDACs leads to a condensed and repressed chromatin state. Thus, HDAC inhibitors produce an increase of histone acetylation and help maintain an open and transcriptionally active DNA [139,140]. To date, 18 HDAC enzymes have been identified, in which the classes of HDAC with respect to their cellular localization and functionalities are summarized in Table 1.

Most tumors possess extensive DNA methylation patterns and increased HDAC expression, causing the epigenetic silencing of tumor-suppressors and immune-stimulatory genes, favoring immune evasion and tumor overgrowth [145]. It is increasingly apparent that epigenetic modulating drugs, such as DNA methyltransferase (DNMT) inhibitors and HDAC inhibitors, can enhance tumor immunogenicity and boosting anti-tumor immunity. Numerous studies have demonstrated synergy between immunotherapies and epigenetic therapy in cancer treatment [146]. DNA demethylating drug 5-aza-2′-deoxycytidine (DAC) testing in an aggressive stroma-rich PDAC mouse model reported the transient inhibition of tumor growth and the retardation of disease progression. The combination of DAC and IFN-γ resulted in an additive anti-proliferative effect on PDAC cells, implying a basis for future studies by combining hypomethylating agents with cytokines and immunotherapy [147]. 

Additionally, HDAC inhibitors have recently been shown to alter the TME immunogenicity of multiple different tumors in several ways, including the upregulating expression of tumor-associated antigens, increasing tumor cell MHC-II expression, inducing NK cell receptor and ligand expression, and decreasing immunosuppressive Tregs and MDSCs [148,149]. Other investigation on the role of HDAC inhibition in immunocompetent murine PDAC model demonstrated that entinostat induces a shift of MDSCs from a myeloid-MDSC-dominant population to a less immunosuppressive G-MDSCs subtype. Moreover, a combinatory regimen of entinostat with immune checkpoint inhibitors (anti-PD1 agent or anti-CTLA-4 antagonist antibody) for PDAC demonstrated a significant improvement in survival compared to the monotherapy of either agents [150].

Of note, the efficacy of epigenetic therapy to modulate the anti-tumor immune response depends on several factors. Firstly, an intact host immune system seems to be the pre-requisite to allow successful epigenetic treatment on cancers [151]. As depicted in the cancer-immunity cycle, generation of an effective anti-tumor immune response requires antigen uptake, processing and presentation to T cells via APCs, followed by the activation of naïve T cells and the trafficking of activated T cells into the tumor for cytolysis [17]. Although HDAC inhibitors can potentially influence each of these steps, the eventual effect of HDAC inhibition on the anti-tumor immunity may vary greatly, depending on the potency and specificity of the HDAC inhibitors used. Furthermore, DNMT inhibitors and HDAC inhibitors are not only effective as single agents, but also reported with much enhanced efficacy when they are used in combination with each other [152]. In fact, current evidence suggests that the most promising potential lies in such incorporation of such epigenetic combination therapies, along with other drugs, such as immunotherapy [153,154]. 

### 5.8. STING Agonists

Activation of the STING pathway can result in the upregulation of type I IFN production. Type I IFNs are not only required for the generation of anti-tumor CD8^+^ T cells but, more importantly, the type 1 interferon transcriptional signature has been linked to “hot”, T-cell-inflamed tumors [155]. In fact, survival advantage was reported in a recent pre-clinical model that employed transgenic mouse model of pancreatic cancer as a treatment target for the STING agonist [156]. The increased production of key inflammatory cytokines and chemokines needed for T-cell migration and the upregulation of maturation markers on DCs, together with an increase in the quantity and functional capacity of the tumor-infiltrating CTLs, was notably present in the tumors, showing that the activation of innate immunity through STING activation can potentially reverse the immunosuppressive PDAC TME [156]. Although radiation only is ineffective at generating systemic antigen-specific immune responses, its combination with novel STING agonists leads to synergistic control of local and distant tumors [157]. This biphasic tumor response is characterized by an initial response that consists of T cell-independent TNFα-driven hemorrhagic necrosis of tumor, followed by a CD8^+^ T-cell-dependent control of residual disease. Immunohistological observation also revealed that STING was abundantly expressed in human pancreatic tumor and stromal cells, but poorly expressed by normal adjacent cells [157]. 

To optimize the anti-tumor immune response, biopolymer devices that allow for the co-delivery of CAR-T cells with STING agonists, which are implantable on solid tumor surfaces, were designed [158]. Once implanted, the co-delivery system of the STING agonists with CAR-T cells can synergistically activate host APCs, besides priming tumor-specific T-cell activation. Following that, the implants were shown to limit the tumor immune escape of inoperable PDAC, apart from eliciting a global anti-tumor immunity, enabling tumor clearance with a modest increase in survival [158]. Taken together, these works demonstrate how innovative approaches can help to optimize effective tumor clearance and systemic anti-tumor immune response. To date, there is one ongoing clinical trial (NCT03010176) that investigates the safety and efficacy profile of STING agonist MK-1454 as monotherapy or in combination with pembrolizumab in several solid tumors and lymphoma. Thus, STING activation is emerging as a potential treatment option in treating PDAC. Nevertheless, the real challenge lies in translating these concepts into a feasible treatment.

## 6. Conclusions

PDAC is a complex disease. Extensive studies have been done and incremental steps have been achieved in understanding this disease entity; however, we have yet to find significant success in treating it. Immunosuppression in PDAC can be driven intrinsically by the tumor cells, as well as through external factors, such as the hosts’ existing immunity and microbiome. Tremendous efforts have been made to develop more effective therapeutic strategies for PDAC; however, it still remains one of the deadliest malignancies with a constantly rising incidence. Although immunotherapy is yet to bring significant successes in treating PDAC, it is still a promising avenue worth exploring. Targeting immune evasion is another emerging strategy targeted to exploit the positive immunomodulatory effects to treat PDAC. We believe that, in time to come, by targeting the signatory PDAC-immune profiles, the greatest determinants of the diagnosis as well as treatment outcome will emerge.

## Figures and Tables

**Table 1:** Summary of Histone deacetylases (HDAC) classes, distribution and general functions.

Class	Members	Cell Location	Functionality/Roles	References
Class I	HDACs 1, 2, 3, 8	Nucleus	Deacetylation of tumor suppressors, steroid receptors, transcription factors	[141]
Class IIA	HDACs 4, 5, 7, 9	Nucleus/cytoplasm	Regulate tissue-specific development and differentiation processes	[141,142]
Class IIB	HDACs 6, 10	Cytoplasm	Regulate signal transduction and motility (via cortactin, HSP90, and tubulin deacetylation)	[141]
Class III (Sirtuins)	SIRT 1–7	Nucleus/cytoplasm/mitochondria	Regulate oxidative stress, DNA repair, metabolism, aging	[141]
Class IV	HDAC 11	Cytoplasm	Oligodendrocyte development, regulate IL-10 by antigen presenting cells	[141,143,144]

## References

[1] Bray F., Ferlay J., Soerjomataram I., Siegel R.L., Torre L.A., Jemal A. (2018). Global cancer statistics 2018: GLOBOCAN estimates of incidence and mortality worldwide for 36 cancers in 185 countries. CA Cancer J. Clin..

[2] American Cancer Society (2020). Cancer Facts and Figures 2020.

[3] Christenson E.S., Jaffee E., Azad N.S. (2020). Current and emerging therapies for patients with advanced pancreatic ductal adenocarcinoma: A bright future. Lancet Oncol..

[4] Increase in Pancreatic Cancer Diagnoses Expected in 2019—Pancreatic Cancer Action Network. https://www.pancan.org/news/increase-in-pancreatic-cancer-diagnoses-expected-in-2019/.

[5] Adamska A., Domenichini A., Falasca M. (2017). Pancreatic ductal adenocarcinoma: Current and evolving therapies. Int. J. Mol. Sci..

[6] Ansari D., Gustafsson A., Andersson R. (2015). Update on the management of pancreatic cancer: Surgery is not enough. World J. Gastroenterol..

[7] Ghosn M., Ibrahim T., Assi T., El Rassy E., Kourie H.R., Kattan J. (2016). Dilemma of first line regimens in metastatic pancreatic adenocarcinoma. World J. Gastroenterol..

[8] Mai C.W., Kang Y.B., Pichika M.R. (2013). Should a Toll-like receptor 4 (TLR-4) agonist or antagonist be designed to treat cancer? TLR-4: Its expression and effects in the ten most common cancers. Onco Targets. Ther..

[9] Mai C.-W., Chung F.F.-L., Leong C.-O. (2017). Targeting legumain as a novel therapeutic strategy in cancers. Curr. Drug Targets.

[10] Mittal D., Gubin M.M., Schreiber R.D., Smyth M.J. (2014). New insights into cancer immunoediting and its three component phases--elimination, equilibrium and escape. Curr. Opin. Immunol..

[11] Looi C.-K., Chung F.F.-L., Leong C.-O., Wong S.-F., Rosli R., Mai C.-W. (2019). Therapeutic challenges and current immunomodulatory strategies in targeting the immunosuppressive pancreatic tumor microenvironment. J. Exp. Clin. Cancer Res..

[12] Collisson E.A., Bailey P., Chang D.K., Biankin A.V. (2019). Molecular subtypes of pancreatic cancer. Nat. Rev. Gastroenterol. Hepatol..

[13] Gajewski T.F., Corrales L., Williams J., Horton B., Sivan A., Spranger S. (2017). Cancer immunotherapy targets based on understanding the T cell-inflamed versus non-T cell-inflamed tumor microenvironment. Adv. Exp. Med. Biol..

[14] Nguyen K.B., Spranger S. (2020). Modulation of the immune microenvironment by tumor-intrinsic oncogenic signaling. J. Cell Biol..

[15] Balkwill F.R., Capasso M., Hagemann T. (2012). The tumor microenvironment at a glance. J. Cell Sci..

[16] Smyth M.J., Dunn G.P., Schreiber R.D. (2006). Cancer immunosurveillance and immunoediting: The roles of immunity in suppressing tumor development and shaping tumor immunogenicity. Adv. Immunol..

[17] Chen D.S., Mellman I. (2013). Oncology meets immunology: The cancer-immunity cycle. Immunity.

[18] Spranger S., Gajewski T.F. (2018). Mechanisms of tumor cell–intrinsic immune evasion. Annu. Rev. Cancer Biol..

[19] Lu L., Zeng J. (2017). Evaluation of K-ras and p53 expression in pancreatic adenocarcinoma using the cancer genome atlas. PLoS ONE.

[20] Xue W., Zender L., Miething C., Dickins R.A., Hernando E., Krizhanovsky V., Cordon-Cardo C., Lowe S.W. (2007). Senescence and tumour clearance is triggered by p53 restoration in murine liver carcinomas. Nature.

[21] Bayne L.J., Beatty G.L., Jhala N., Clark C.E., Rhim A.D., Stanger B.Z., Vonderheide R.H. (2012). Tumor-Derived Granulocyte-Macrophage Colony-Stimulating Factor Regulates Myeloid Inflammation and T Cell Immunity in Pancreatic Cancer. Cancer Cell.

[22] Pylayeva-Gupta Y., Lee K.E., Hajdu C.H., Miller G., Bar-Sagi D. (2012). Oncogenic Kras-Induced GM-CSF Production Promotes the Development of Pancreatic Neoplasia. Cancer Cell.

[23] Blagih J., Zani F., Chakravarty P., Hennequart M., Pilley S., Hobor S., Hock A.K., Walton J.B., Morton J.P., Gronroos E. (2020). Cancer-specific loss of p53 leads to a modulation of myeloid and T cell responses. Cell Rep..

[24] Iannello A., Thompson T.W., Ardolino M., Lowe S.W., Raulet D.H. (2013). p53-dependent chemokine production by senescent tumor cells supports NKG2D-dependent tumor elimination by natural killer cells. J. Exp. Med..

[25] Guo G., Yu M., Xiao W., Celis E., Cui Y. (2017). Local activation of p53 in the tumor microenvironment overcomes immune suppression and enhances antitumor immunity. Cancer Res..

[26] Shackelford D.B., Shaw R.J. (2009). The LKB1-AMPK pathway: Metabolism and growth control in tumour suppression. Nat. Rev. Cancer.

[27] Yang J.Y., Jiang S.H., Liu D.J., Yang X.M., Huo Y.M., Li J., Hua R., Zhang Z.G., Sun Y.W. (2015). Decreased LKB1 predicts poor prognosis in pancreatic ductal adenocarcinoma. Sci. Rep..

[28] Koyama S., Akbay E.A., Li Y.Y., Aref A.R., Skoulidis F., Herter-Sprie G.S., Buczkowski K.A., Liu Y., Awad M.M., Denning W.L. (2016). STK11/LKB1 deficiency promotes neutrophil recruitment and proinflammatory cytokine production to suppress T-cell activity in the lung tumor microenvironment. Cancer Res..

[29] Kitajima S., Ivanova E., Guo S., Yoshida R., Campisi M., Sundararaman S.K., Tange S., Mitsuishi Y., Thai T.C., Masuda S. (2019). Suppression of STING associated with lkb1 loss in KRAS-driven lung cancer. Cancer Discov..

[30] Nguyen H.B., Babcock J.T., Wells C.D., Quilliam L.A. (2013). LKB1 tumor suppressor regulates AMP kinase/mTOR-independent cell growth and proliferation via the phosphorylation of Yap. Oncogene.

[31] Murakami S., Shahbazian D., Surana R., Zhang W., Chen H., Graham G.T., White S.M., Weiner L.M., Yi C. (2017). Yes-Associated protein mediates immune reprogramming in pancreatic ductal adenocarcinoma. Oncogene.

[32] Flavahan W.A., Gaskell E., Bernstein B.E. (2017). Epigenetic plasticity and the hallmarks of cancer. Science.

[33] Jung H., Kim H.S., Kim J.Y., Sun J.M., Ahn J.S., Ahn M.J., Park K., Esteller M., Lee S.H., Choi J.K. (2019). DNA methylation loss promotes immune evasion of tumours with high mutation and copy number load. Nat. Commun..

[34] Deicher A., Andersson R., Tingstedt B., Lindell G., Bauden M., Ansari D. (2018). Targeting dendritic cells in pancreatic ductal adenocarcinoma. Cancer Cell Int..

[35] Cruickshank B., Giacomantonio M., Marcato P., McFarland S., Pol J., Gujar S. (2018). Dying to be noticed: Epigenetic regulation of immunogenic cell death for cancer immunotherapy. Front. Immunol..

[36] Lu C., Paschall A.V., Shi H., Savage N., Waller J.L., Sabbatini M.E., Oberlies N.H., Pearce C., Liu K. (2017). The MLL1-H3K4me3 Axis-Mediated PD-L1 Expression and Pancreatic Cancer Immune Evasion. J. Natl. Cancer Inst..

[37] Shen W.H., Balajee A.S., Wang J., Wu H., Eng C., Pandolfi P.P., Yin Y. (2007). Essential Role for Nuclear PTEN in Maintaining Chromosomal Integrity. Cell.

[38] Brandmaier A., Hou S.Q., Demaria S., Formenti S.C., Shen W.H. (2017). PTEN at the interface of immune tolerance and tumor suppression. Front. Biol. (Beijing).

[39] Ying H., Elpek K.G., Vinjamoori A., Zimmerman S.M., Chu G.C., Yan H., Fletcher-Sananikone E., Zhang H., Liu Y., Wang W. (2011). PTEN is a major tumor suppressor in pancreatic ductal adenocarcinoma and regulates an NF-κB-cytokine network. Cancer Discov..

[40] Dong Y., Richards J.A., Gupta R., Aung P.P., Emley A., Kluger Y., Dogra S.K., Mahalingam M., Wajapeyee N. (2014). PTEN functions as a melanoma tumor suppressor by promoting host immune response. Oncogene.

[41] Waldron J.S., Yang I., Han S., Tihan T., Sughrue M.E., Mills S.A., Pieper R.O., Parsa A.T. (2010). Implications for immunotherapy of tumor-mediated T-cell apoptosis associated with loss of the tumor suppressor PTEN in glioblastoma. J. Clin. Neurosci..

[42] Ren W.H., Zhang X.R., Li W.B., Feng Q., Feng H.J., Tong Y., Rong H., Wang W., Zhang D., Zhang Z.Q. (2019). Exosomal miRNA-107 induces myeloid-derived suppressor cell expansion in gastric cancer. Cancer Manag. Res..

[43] Peng W., Chen J.Q., Liu C., Malu S., Creasy C., Tetzlaff M.T., Xu C., McKenzie J.A., Zhang C., Liang X. (2016). Loss of PTEN promotes resistance to T cell–mediated immunotherapy. Cancer Discov..

[44] Reya T., Clevers H. (2005). Wnt signalling in stem cells and cancer. Nature.

[45] Esteller M., Sparks A., Toyota M., Sanchez-Cespedes M., Capella G., Peinado M.A., Gonzalez S., Tarafa G., Sidransky D., Meltzer S.J. (2000). Analysis of adenomatous polyposis coli promoter hypermethylation in human cancer. Cancer Res..

[46] Jiang X., Cao Y., Li F., Su Y., Li Y., Peng Y., Cheng Y., Zhang C., Wang W., Ning G. (2014). Targeting i 2-catenin signaling for therapeutic intervention in MEN1-deficient pancreatic neuroendocrine tumours. Nat. Commun..

[47] Jiang X., Hao H.X., Growney J.D., Woolfenden S., Bottiglio C., Ng N., Lu B., Hsieh M.H., Bagdasarian L., Meyer R. (2013). Inactivating mutations of RNF43 confer Wnt dependency in pancreatic ductal adenocarcinoma. Proc. Natl. Acad. Sci. USA.

[48] Tang B., Yang Y., Kang M., Wang Y., Wang Y., Bi Y., He S., Shimamoto F. (2020). M6A demethylase ALKBH5 inhibits pancreatic cancer tumorigenesis by decreasing WIF-1 RNA methylation and mediating Wnt signaling. Mol. Cancer.

[49] Igbinigie E., Guo F., Jiang S.W., Kelley C., Li J. (2019). Dkk1 involvement and its potential as a biomarker in pancreatic ductal adenocarcinoma. Clin. Chim. Acta.

[50] Luke J.J., Bao R., Sweis R.F., Spranger S., Gajewski T.F. (2019). WNT/b-catenin pathway activation correlates with immune exclusion across human cancers. Clin. Cancer Res..

[51] Argentiero A., De Summa S., Di Fonte R., Iacobazzi R.M., Porcelli L., Da Vià M., Brunetti O., Azzariti A., Silvestris N., Solimando A.G. (2019). Gene expression comparison between the lymph node-positive and -negative reveals a peculiar immune microenvironment signature and a theranostic role for WNT targeting in pancreatic ductal adenocarcinoma: A pilot study. Cancers (Basel).

[52] Joffre O.P., Segura E., Savina A., Amigorena S. (2012). Cross-presentation by dendritic cells. Nat. Rev. Immunol..

[53] Heiden M.G.V., Cantley L.C., Thompson C.B. (2009). Understanding the warburg effect: The metabolic requirements of cell proliferation. Science.

[54] Itakura J., Ishiwata T., Friess H., Fujii H., Matsumoto Y., Büchler M.W., Korc M. (1997). Enhanced expression of vascular endothelial growth factor in human pancreatic cancer correlates with local disease progression. Clin. Cancer Res..

[55] Daniel S.K., Sullivan K.M., Labadie K.P., Pillarisetty V.G. (2019). Hypoxia as a barrier to immunotherapy in pancreatic adenocarcinoma. Clin. Transl. Med..

[56] Bharadwaj U., Li M., Zhang R., Chen C., Yao Q. (2007). Elevated interleukin-6 and G-CSF in human pancreatic cancer cell conditioned medium suppress dendritic cell differentiation and activation. Cancer Res..

[57] Bausch D., Pausch T., Krauss T., Hopt U.T., Fernandez-Del-Castillo C., Warshaw A.L., Thayer S.P., Keck T. (2011). Neutrophil granulocyte derived MMP-9 is a VEGF independent functional component of the angiogenic switch in pancreatic ductal adenocarcinoma. Angiogenesis.

[58] Lu Y., Hu J., Sun W., Duan X., Chen X. (2015). Hypoxia-mediated immune evasion of pancreatic carcinoma cells. Mol. Med. Rep..

[59] Ou Z.L., Luo Z., Wei W., Liang S., Gao T.L., Lu Y. (2019). Bin Hypoxia-induced shedding of MICA and HIF1A-mediated immune escape of pancreatic cancer cells from NK cells: Role of circ_0000977/miR-153 axis. RNA Biol..

[60] Attri K.S., Mehla K., Singh P.K. (2018). Evaluation of macrophage polarization in pancreatic cancer microenvironment under hypoxia. Methods in Molecular Biology.

[61] Song J., Lee J., Kim J., Jo S., Kim Y.J., Baek J.E., Kwon E.S., Lee K.P., Yang S., Kwon K.S. (2016). Pancreatic adenocarcinoma up-regulated factor (PAUF) enhances the accumulation and functional activity of myeloidderived suppressor cells (MDSCs) in pancreatic cancer. Oncotarget.

[62] Feig C., Gopinathan A., Neesse A., Chan D.S., Cook N., Tuveson D.A. (2012). The pancreas cancer microenvironment. Clin. Cancer Res..

[63] Jiang H., Hegde S., Knolhoff B.L., Zhu Y., Herndon J.M., Meyer M.A., Nywening T.M., Hawkins W.G., Shapiro I.M., Weaver D.T. (2016). Targeting focal adhesion kinase renders pancreatic cancers responsive to checkpoint immunotherapy. Nat. Med..

[64] Torphy R.J., Zhu Y., Schulick R.D. (2018). Immunotherapy for pancreatic cancer: Barriers and breakthroughs. Ann. Gastroenterol. Surg..

[65] Parsons J.T., Slack-Davis J., Tilghman R., Roberts W.G. (2008). Focal adhesion kinase: Targeting adhesion signaling pathways for therapeutic intervention. Clin. Cancer Res..

[66] Laklai H., Miroshnikova Y.A., Pickup M.W., Collisson E.A., Kim G.E., Barrett A.S., Hill R.C., Lakins J.N., Schlaepfer D.D., Mouw J.K. (2016). Genotype tunes pancreatic ductal adenocarcinoma tissue tension to induce matricellular fibrosis and tumor progression. Nat. Med..

[67] Korc M. (2018). Beyond Kras: MYC rules in pancreatic cancer. Cell Mol. Gastroenterol. Hepatol..

[68] Sodir N.M., Kortlever R.M., Barthet V.J.A., Campos T., Pellegrinet L., Kupczak S., Anastasiou P., Swigart L.B., Soucek L., Arends M.J. (2020). MYC Instructs and Maintains Pancreatic Adenocarcinoma Phenotype. Cancer Discov..

[69] Casey S.C., Tong L., Li Y., Do R., Walz S., Fitzgerald K.N., Gouw A.M., Baylot V., Gütgemann I., Eilers M. (2016). MYC regulates the antitumor immune response through CD47 and PD-L1. Science.

[70] Cioffi M., Trabulo S., Hidalgo M., Costello E., Greenhalf W., Erkan M., Kleeff J., Sainz B., Heeschen C. (2015). Cancer Therapy: Preclinical inhibition of CD47 effectively targets pancreatic cancer stem cells via dual mechanisms. Clin. Cancer Res..

[71] Dunn G.P., Bruce A.T., Ikeda H., Old L.J., Schreiber R.D. (2002). Cancer immunoediting: From immunosurveillance to tumor escape. Nat. Immunol..

[72] Dupage M., Mazumdar C., Schmidt L.M., Cheung A.F., Jacks T. (2012). Expression of tumour-specific antigens underlies cancer immunoediting. Nature.

[73] Dunn G.P., Old L.J., Schreiber R.D. (2004). The three Es of cancer immunoediting. Annu. Rev. Immunol..

[74] Coulie P.G., Van Den Eynde B.J., Van Der Bruggen P., Boon T. (2014). Tumour antigens recognized by T lymphocytes: At the core of cancer immunotherapy. Nat. Rev. Cancer.

[75] Peterson A.C., Harlin H., Gajewski T.F. (2003). Immunization with melan-A peptide-pulsed peripheral blood mononuclear cells plus recombinant human interleukin-12 induces clinical activity and T-cell responses in advanced melanoma. J. Clin. Oncol..

[76] Evans R.A., Diamond M.S., Rech A.J., Chao T., Richardson M.W., Lin J.H., Bajor D.L., Byrne K.T., Stanger B.Z., Riley J.L. (2016). Lack of immunoediting in murine pancreatic cancer reversed with neoantigen. JCI Insight.

[77] Vonderheide R.H., Bayne L.J. (2013). Inflammatory networks and immune surveillance of pancreatic carcinoma. Curr. Opin. Immunol..

[78] Müller M., Briscoe J., Laxton C., Guschin D., Ziemiecki A., Silvennoinen O., Harpur A.G., Barbieri G., Witthuhn B.A., Schindler C. (1993). The protein tyrosine kinase JAK1 complements defects in interferon-α/β and -γ Signal transduction. Nature.

[79] Bach E.A., Aguet M., Schreiber R.D. (1997). The IFN Gamma Receptor:A Paradigm for Cytokine Receptor Signaling. Annu. Rev. Immunol..

[80] Rosenberg J.E., Hoffman-Censits J., Powles T., Van Der Heijden M.S., Balar A.V., Necchi A., Dawson N., O’Donnell P.H., Balmanoukian A., Loriot Y. (2016). Atezolizumab in patients with locally advanced and metastatic urothelial carcinoma who have progressed following treatment with platinum-based chemotherapy: A single-arm, multicentre, phase 2 trial. Lancet.

[81] Chiang A.C., Herbst R.S. (2020). Frontline immunotherapy for NSCLC—The tale of the tail. Nat. Rev. Clin. Oncol..

[82] Brahmer J.R., Tykodi S.S., Chow L.Q.M., Hwu W.J., Topalian S.L., Hwu P., Drake C.G., Camacho L.H., Kauh J., Odunsi K. (2012). Safety and activity of anti-PD-L1 antibody in patients with advanced cancer. N. Engl. J. Med..

[83] Hodi F.S., O’Day S.J., McDermott D.F., Weber R.W., Sosman J.A., Haanen J.B., Gonzalez R., Robert C., Schadendorf D., Hassel J.C. (2010). Improved survival with ipilimumab in patients with metastatic melanoma. N. Engl. J. Med..

[84] Freeman G.J., Long A.J., Iwai Y., Bourque K., Chernova T., Nishimura H., Fitz L.J., Malenkovich N., Okazaki T., Byrne M.C. (2000). Engagement of the PD-1 immunoinhibitory receptor by a novel B7 family member leads to negative regulation of lymphocyte activation. J. Exp. Med..

[85] Robert C., Long G.V., Brady B., Dutriaux C., Maio M., Mortier L., Hassel J.C., Rutkowski P., McNeil C., Kalinka-Warzocha E. (2015). Nivolumab in previously untreated melanoma without *BRAF* mutation. N. Engl. J. Med..

[86] Reck M., Rodriguez-Abreu D., Robinson A.G., Hui R., Csöszi T., Fülöp A., Gottfried M., Peled N., Tafreshi A., Cuffe S. (2016). Pembrolizumab versus chemotherapy for PD-L1-positive non-small-cell lung cancer. N. Engl. J. Med..

[87] Nomi T., Sho M., Akahori T., Hamada K., Kubo A., Kanehiro H., Nakamura S., Enomoto K., Yagita H., Azuma M. (2007). Clinical significance and therapeutic potential of the programmed death-1 ligand/programmed death-1 pathway in human pancreatic cancer. Clin. Cancer Res..

[88] Yamaki S., Yanagimoto H., Tsuta K., Ryota H., Kon M. (2017). PD-L1 expression in pancreatic ductal adenocarcinoma is a poor prognostic factor in patients with high CD8+ tumor-infiltrating lymphocytes: Highly sensitive detection using phosphor-integrated dot staining. Int. J. Clin. Oncol..

[89] Leach D.R., Krummel M.F., Allison J.P. (1996). Enhancement of antitumor immunity by CTLA-4 blockade. Science.

[90] Royal R.E., Levy C., Turner K., Mathur A., Hughes M., Kammula U.S., Sherry R.M., Topalian S.L., Yang J.C., Lowy I. (2010). Phase 2 Trial of Single Agent Ipilimumab (Anti-CTLA-4) for Locally Advanced or Metastatic Pancreatic Adenocarcinoma. J. Immunother..

[91] Sharma P., Dirix L., De Vos F.Y.F.L., Allison J.P., Decoster L., Zaucha R., Park J.O., Vanderwalde A.M., Kataria R.S., Ferro S. (2018). Efficacy and tolerability of tremelimumab in patients with metastatic pancreatic ductal adenocarcinoma. J. Clin. Oncol..

[92] Le D.T., Durham J.N., Smith K.N., Wang H., Bartlett B.R., Aulakh L.K., Lu S., Kemberling H., Wilt C., Luber B.S. (2017). Mismatch repair deficiency predicts response of solid tumors to PD-1 blockade. Science.

[93] Humphris J.L., Patch A.M., Nones K., Bailey P.J., Johns A.L., McKay S., Chang D.K., Miller D.K., Pajic M., Kassahn K.S. (2017). Hypermutation In Pancreatic Cancer. Gastroenterology.

[94] Zhu Y., Knolhoff B.L., Meyer M.A., Nywening T.M., West B.L., Luo J., Wang-Gillam A., Goedegebuure S.P., Linehan D.C., De Nardo D.G. (2014). CSF1/CSF1R blockade reprograms tumor-infiltrating macrophages and improves response to T-cell checkpoint immunotherapy in pancreatic cancer models. Cancer Res..

[95] Er J.L., Goh P.N., Lee C.Y., Tan Y.J., Hii L.-W., Mai C.W., Chung F.F.-L., Leong C.-O. (2018). Identification of inhibitors synergizing gemcitabine sensitivity in the squamous subtype of pancreatic ductal adenocarcinoma (PDAC). Apoptosis.

[96] Hii L.-W., Lim S.-H.E., Leong C.-O., Chin S.-Y., Tan N.-P., Lai K.-S., Mai C.-W. (2019). The synergism of *Clinacanthus nutans* Lindau extracts with gemcitabine: Downregulation of anti-apoptotic markers in squamous pancreatic ductal adenocarcinoma. BMC Complement. Altern. Med..

[97] Cassier P.A., Garin G., Eberst L., Delord J.-P., Chabaud S., Terret C., Montane L., Bidaux A.-S., Laurent S., Jaubert L. (2019). MEDIPLEX: A phase 1 study of durvalumab (D) combined with pexidartinib (P) in patients (pts) with advanced pancreatic ductal adenocarcinoma (PDAC) and colorectal cancer (CRC). J. Clin. Oncol..

[98] Deng L., Liang H., Burnette B., Beckett M., Darga T., Weichselbaum R.R., Fu Y.X. (2014). Irradiation and anti-PD-L1 treatment synergistically promote antitumor immunity in mice. J. Clin. Investig..

[99] Kumar S., Principe D., Singh S., Viswakarma N., Sondarva G., Rana B., Rana A. (2020). Mitogen-Activated Protein Kinase Inhibitors and T-Cell-Dependent Immunotherapy in Cancer. Pharmaceuticals.

[100] Ebert P.J.R., Cheung J., Yang Y., McNamara E., Hong R., Moskalenko M., Gould S.E., Maecker H., Irving B.A., Kim J.M. (2016). MAP kinase inhibition promotes T cell and anti-tumor activity in combination with PD-L1 checkpoint blockade. Immunity.

[101] Alagesan B., Contino G., Guimaraes A.R., Corcoran R.B., Deshpande V., Wojtkiewicz G.R., Hezel A.F., Wong K.K., Loda M., Weissleder R. (2015). Combined MEK and PI3K inhibition in a mouse model of pancreatic cancer. Clin. Cancer Res..

[102] Soo H.-C., Chung F.F.-L., Lim K.-H., Yap V.A., Bradshaw T.D., Hii L.-W., Tan S.-H., See S.-J., Tan Y.-F., Leong C.-O. (2017). Cudraflavone C induces tumor-specific apoptosis in colorectal cancer cells through inhibition of the phosphoinositide 3-kinase (PI3K)-AKT pathway. PLoS ONE.

[103] Yang S., Wang X., Contino G., Liesa M., Sahin E., Ying H., Bause A., Li Y., Stomme J.M., Dell’Antonio G. (2011). Pancreatic cancers require autophagy for tumor growth. Genes Dev..

[104] Yang A., Herter-Sprie G., Zhang H., Lin E.Y., Biancur D., Wang X., Deng J., Hai J., Yang S., Wong K.K. (2018). Autophagy sustains pancreatic cancer growth through both cell-autonomous and nonautonomous mechanisms. Cancer Discov..

[105] Yamamoto K., Venida A., Yano J., Biancur D.E., Kakiuchi M., Gupta S., Sohn A.S.W., Mukhopadhyay S., Lin E.Y., Parker S.J. (2020). Autophagy promotes immune evasion of pancreatic cancer by degrading MHC-I. Nature.

[106] Noman M.Z., Parpal S., Van Moer K., Xiao M., Yu Y., Viklund J., De Milito A., Hasmim M., Andersson M., Amaravadi R.K. (2020). Inhibition of Vps34 reprograms cold into hot inflamed tumors and improves anti-PD-1/PD-L1 immunotherapy. Sci. Adv..

[107] Azad A., Yin Lim S., D’Costa Z., Jones K., Diana A., Sansom O.J., Kruger P., Liu S., McKenna W.G., Dushek O. (2017). PD-L1 blockade enhances response of pancreatic ductal adenocarcinoma to radiotherapy. EMBO Mol. Med..

[108] Le D.T., Picozzi V.J., Ko A.H., Wainberg Z.A., Kindler H., Wang-Gillam A., Oberstein P., Morse M.A., Zeh H.J., Weekes C. (2019). Results from a phase IIb, randomized, multicenter study of GVAX pancreas and CRS-207 compared with chemotherapy in adults with previously treated metastatic pancreatic adenocarcinoma (ECLIPSE study). Clin. Cancer Res..

[109] Eric L., Yeo C.J., Lillemoe K.D., Biedrzycki B., Kobrin B., Herman J., Sugar E., Piantadosi S., Cameron J.L., Solt S. (2011). A lethally irradiated allogeneic granulocyte-macrophage colony stimulating factor-secreting tumor vaccine for pancreatic adenocarcinoma: A phase II trial of safety, efficacy, and immune activation. Ann. Surg..

[110] NewLink Geneticsannounces Results from Phase 3 IMPRESS Trial of Algenpantucel-L for Patients with Resected Pancreatic Cancer Nasdaq: NLNK. https://www.globenewswire.com/news-release/2016/05/09/837878/0/en/NewLink-Genetics-Announces-Results-from-Phase-3-IMPRESS-Trial-of-Algenpantucel-L-for-Patients-with-Resected-Pancreatic-Cancer.html.

[111] Middleton G., Silcocks P., Cox T., Valle J., Wadsley J., Propper D., Coxon F., Ross P., Madhusudan S., Roques T. (2014). Gemcitabine and capecitabine with or without telomerase peptide vaccine GV1001 in patients with locally advanced or metastatic pancreatic cancer (TeloVac): An open-label, randomised, phase 3 trial. Lancet Oncol..

[112] Bernhardt S.L., Gjertsen M.K., Trachsel S., Møller M., Eriksen J.A., Meo M., Buanes T., Gaudernack G. (2006). Telomerase peptide vaccination of patients with non-resectable pancreatic cancer: A dose escalating phase I/II study. Br. J. Cancer.

[113] Hinrichs C.S., Rosenberg S.A. (2014). Exploiting the curative potential of adoptive T-cell therapy for cancer. Immunol. Rev..

[114] Wang X., Rivière I. (2016). Clinical manufacturing of CAR T cells: Foundation of a promising therapy. Mol. Ther. Oncolytics.

[115] Ali A.I., Oliver A.J., Samiei T., Chan J.D., Kershaw M.H., Slaney C.Y. (2019). Genetic redirection of T cells for the treatment of pancreatic cancer. Front. Oncol..

[116] Beatty G.L., O’Hara M.H., Lacey S.F., Torigian D.A., Nazimuddin F., Chen F., Kulikovskaya I.M., Soulen M.C., McGarvey M., Nelson A.M. (2018). Activity of mesothelin-specific chimeric antigen receptor T cells against pancreatic carcinoma metastases in a phase 1 trial. Gastroenterology.

[117] Jiang H., Shi Z., Wang P., Wang C., Yang L., Du G., Zhang H., Shi B., Jia J., Li Q. (2019). Claudin18.2-specific chimeric antigen receptor engineered T cells for the treatment of gastric cancer. J. Natl. Cancer Inst..

[118] Preliminary Data with CAR-Claudin18.2-T in Gastric and Pancreatic Cancer. https://medi-paper.com/car-tcr-summit-2018-preliminary-first-in-human-data-with-car-claudin18-2-t-in-gastric-and-pancreatic-cancer/.

[119] Kaufman H.L., Kohlhapp F.J., Zloza A. (2015). Oncolytic viruses: A new class of immunotherapy drugs. Nat. Rev. Drug Discov..

[120] Gujar S., Pol J.G., Kim Y., Lee P.W., Kroemer G. (2018). Antitumor benefits of antiviral immunity: An underappreciated aspect of oncolytic virotherapies. Trends Immunol..

[121] Lichty B.D., Breitbach C.J., Stojdl D.F., Bell J.C. (2014). Going viral with cancer immunotherapy. Nat. Rev. Cancer.

[122] Mahalingam D., Wilkinson G.A., Eng K.H., Fields P., Raber P., Moseley J.L., Cheetham K., Coffey M., Nuovo G., Kalinski P. (2020). Pembrolizumab in combination with the oncolytic virus pelareorep and chemotherapy in patients with advanced pancreatic adenocarcinoma: A phase Ib study. Clin. Cancer Res..

[123] Hashimoto Y., Ueno M., Tanaka M., Ikeda M. (2019). Results from phase I study of the oncolytic viral immunotherapy agent canerpaturev (C-REV) in combination with gemcitabine plus nab-paclitaxel for unresectable pancreatic cancer. J. Clin. Oncol..

[124] Wein L.M., Wu J.T., Kirn D.H. (2003). Validation and analysis of a mathematical model of a replication-competent oncolytic virus for cancer treatment: Implications for virus design and delivery. Cancer Res..

[125] Wojton J., Kaur B. (2010). Impact of tumor microenvironment on oncolytic viral therapy. Cytokine Growth Factor Rev..

[126] Ferguson M.S., Lemoine N.R., Wang Y. (2012). Systemic delivery of oncolytic viruses: Hopes and hurdles. Adv. Virol..

[127] García-Castro J., Alemany R., Cascalló M., Martínez-Quintanilla J., Del Mar Arriero M., Lassaletta Á., Madero L., Ramírez M. (2010). Treatment of metastatic neuroblastoma with systemic oncolytic virotherapy delivered by autologous mesenchymal stem cells: An exploratory study. Cancer Gene Ther..

[128] Guo Z.S., Parimi V., O’Malley M.E., Thirunavukarasu P., Sathaiah M., Austin F., Bartlett D.L. (2010). The combination of immunosuppression and carrier cells significantly enhances the efficacy of oncolytic poxvirus in the pre-immunized host. Gene Ther..

[129] Raki M., Sarkioja M., Escutenaire S., Kangasniemi L., Haavisto E., Kanerva A., Cerullo V., Joensuu T., Oksanen M., Pesonen S. (2011). Switching the fiber knob of oncolytic adenoviruses to avoid neutralizing antibodies in human cancer patients. J. Gene Med..

[130] Vonderheide R.H. (2007). Prospect of targeting the CD40 pathway for cancer therapy. Clin. Cancer Res..

[131] Grewal I.S., Flavell R.A. (1998). CD40 and CD154 in cell-mediated immunity. Annu. Rev. Immunol..

[132] Beatty G.L., Torigian D.A., Gabriela Chiorean E., Saboury B., Brothers A., Alavi A., Troxel A.B., Sun W., Teitelbaum U.R., Vonderheide R.H. (2013). A phase I study of an agonist CD40 monoclonal antibody (CP-870,893) in combination with gemcitabine in patients with advanced pancreatic ductal adenocarcinoma. Clin. Cancer Res..

[133] Vonderheide R.H. (2020). CD40 agonist antibodies incancer immunotherapy. Annu. Rev. Med..

[134] Moon Y.W., Hajjar J., Hwu P., Naing A. (2015). Targeting the indoleamine 2,3-dioxygenase pathway in cancer. J. Immunother. Cancer.

[135] Bahary N., Garrido-Laguna I., Cinar P., Somer B.G., Nayak-Kapoor A., Lee J.S., Munn D., Kennedy E., Vahanian N.N., Link C.J. (2018). Phase 2 trial of the indoleamine 2,3-dioxygenase pathway (IDO) inhibitor indoximod plus gemcitabine/nab-paclitaxel for the treatment of metastatic pancreas cancer. J Clin. Oncol..

[136] Muller A.J., DuHadaway J.B., Donover P.S., Sutanto-Ward E., Prendergast G.C. (2005). Inhibition of indoleamine 2,3-dioxygenase, an immunoregulatory target of the cancer suppression gene Bin1, potentiates cancer chemotherapy. Nat. Med..

[137] Soliman H.H., Jackson E., Neuger T., Claire Dees E., Donald Harvey R., Han H., Ismail-Khan R., Minton S., Vahanian N.N., Link C. (2014). A first in man phase I trial of the oral immunomodulator, indoximod, combined with docetaxel in patients with metastatic solid tumors. Oncotarget.

[138] Zakharia Y., Rixe O., Ward J.H., Drabick J.J., Shaheen M.F., Milhem M.M., Munn D., Kennedy E.P., Vahanian N.N., Link C.J. (2018). Phase 2 trial of the IDO pathway inhibitor indoximod plus checkpoint inhibition for the treatment of patients with advanced melanoma. J. Clin. Oncol..

[139] Drummond D.C., Noble C.O., Kirpotin D.B., Guo Z., Scott G.K., Benz C.C. (2005). Clinical development of histone deacetylase inhibitors as anticancer agents. Annu. Rev. Pharmacol. Toxicol..

[140] Minucci S., Pelicci P.G. (2006). Histone deacetylase inhibitors and the promise of epigenetic (and more) treatments for cancer. Nat. Rev. Cancer.

[141] Hull E.E., Montgomery M.R., Leyva K.J. (2016). HDAC inhibitors as epigenetic regulators of the immune system: Impacts on cancer therapy and inflammatory diseases. Biomed Res. Int..

[142] Parra M. (2015). Class IIa HDACs—New insights into their functions in physiology and pathology. FEBS J..

[143] Liu H., Hu Q., D’ercole A.J., Ye P. (2009). Histone deacetylase 11 regulates oligodendrocyte-specific gene expression and cell development in OL-1 oligodendroglia cells. Glia.

[144] Villagra A., Cheng F., Wang H.W., Suarez I., Glozak M., Maurin M., Nguyen D., Wright K.L., Atadja P.W., Bhalla K. (2009). The histone deacetylase HDAC11 regulates the expression of interleukin 10 and immune tolerance. Nat. Immunol..

[145] Schreiber R.D., Old L.J., Smyth M.J. (2011). Cancer immunoediting: Integrating immunity’s roles in cancer suppression and promotion. Science.

[146] Aspeslagh S., Morel D., Soria J.C., Postel-Vinay S. (2018). Epigenetic modifiers as new immunomodulatory therapies in solid tumours. Ann. Oncol..

[147] Shakya R., Gonda T., Quante M., Salas M., Kim S., Brooks J., Hirsch S., Davies J., Cullo A., Olive K. (2013). Hypomethylating therapy in an aggressive stroma-rich model of pancreatic carcinoma. Cancer Res..

[148] Zhu S., Denman C.J., Cobanoglu Z.S., Kiany S., Lau C.C., Gottschalk S.M., Hughes D.P.M., Kleinerman E.S., Lee D.A. (2015). The narrow-spectrum HDAC inhibitor entinostat enhances NKG2D expression without NK cell toxicity, leading to enhanced recognition of cancer cells. Pharm. Res..

[149] Cycon K.A., Mulvaney K., Rimsza L.M., Persky D., Murphy S.P. (2013). Histone deacetylase inhibitors activate CIITA and MHC class II antigen expression in diffuse large B-cell lymphoma. Immunology.

[150] Christmas B.J., Rafie C.I., Hopkins A.C., Scott B.A., Ma H.S., Cruz K.A., Woolman S., Armstrong T.D., Connolly R.M., Azad N.A. (2018). Entinostat converts immune-resistant breast and pancreatic cancers into checkpoint-responsive tumors by reprogramming tumor-infiltrating MDSCs. Cancer Immunol. Res..

[151] West A.C., Mattarollo S.R., Shortt J., Cluse L.A., Christiansen A.J., Smyth M.J., Johnstone R.W. (2013). An intact immune system is required for the anticancer activities of histone deacetylase inhibitors. Cancer Res..

[152] Roberti A., Valdes A.F., Torrecillas R., Fraga M.F., Fernandez A.F. (2019). Epigenetics in cancer therapy and nanomedicine. Clin. Epigenet..

[153] Juergens R.A., Wrangle J., Vendetti F.P., Murphy S.C., Zhao M., Coleman B., Sebree R., Rodgers K., Hooker C.M., Franco N. (2011). Combination epigenetic therapy has efficacy in patients with refractory advanced non-small cell lung cancer. Cancer Discov..

[154] Chiappinelli K.B., Zahnow C.A., Ahuja N., Baylin S.B. (2016). Combining epigenetic and immunotherapy to combat cancer. Cancer Res..

[155] Fuertes M.B., Kacha A.K., Kline J., Woo S.R., Kranz D.M., Murphy K.M., Gajewski T.F. (2011). Host type I IFN signals are required for antitumor CD8+ T cell responses through CD8α+ dendritic cells. J. Exp. Med..

[156] Jing W., McAllister D., Vonderhaar E.P., Palen K., Riese M.J., Gershan J., Johnson B.D., Dwinell M.B. (2019). STING agonist inflames the pancreatic cancer immune microenvironment and reduces tumor burden in mouse models. J. Immunother. Cancer.

[157] Baird J.R., Friedman D., Cottam B., Dubensky T.W., Kanne D.B., Bambina S., Bahjat K., Crittenden M.R., Gough M.J. (2016). Radiotherapy combined with novel STING-targeting oligonucleotides results in regression of established tumors. Cancer Res..

[158] Smith T.T., Moffett H.F., Stephan S.B., Opel C.F., Dumigan A.G., Jiang X., Pillarisetty V.G., Pillai S.P.S., Wittrup K.D., Stephan M.T. (2017). Biopolymers codelivering engineered T cells and STING agonists can eliminate heterogeneous tumors. J. Clin. Investig..

